# Cloning large natural product gene clusters from the environment: Piecing environmental DNA gene clusters back together with TAR

**DOI:** 10.1002/bip.21450

**Published:** 2010-09

**Authors:** Jeffrey H Kim, Zhiyang Feng, John D Bauer, Dimitris Kallifidas, Paula Y Calle, Sean F Brady

**Affiliations:** Howard Hughes Medical Institute, Laboratory of Genetically Encoded Small Molecules, The Rockefeller University1230 York Avenue, New York, NY 10065

**Keywords:** natural products, eDNA, metagenomics, TAR

## Abstract

A single gram of soil can contain thousands of unique bacterial species, of which only a small fraction is regularly cultured in the laboratory. Although the fermentation of cultured microorganisms has provided access to numerous bioactive secondary metabolites, with these same methods it is not possible to characterize the natural products encoded by the uncultured majority. The heterologous expression of biosynthetic gene clusters cloned from DNA extracted directly from environmental samples (eDNA) has the potential to provide access to the chemical diversity encoded in the genomes of uncultured bacteria. One of the challenges facing this approach has been that many natural product biosynthetic gene clusters are too large to be readily captured on a single fragment of cloned eDNA. The reassembly of large eDNA-derived natural product gene clusters from collections of smaller overlapping clones represents one potential solution to this problem. Unfortunately, traditional methods for the assembly of large DNA sequences from multiple overlapping clones can be technically challenging. Here we present a general experimental framework that permits the recovery of large natural product biosynthetic gene clusters on overlapping soil-derived eDNA cosmid clones and the reassembly of these large gene clusters using transformation-associated recombination (TAR) in *Saccharomyces cerevisiae*. The development of practical methods for the rapid assembly of biosynthetic gene clusters from collections of overlapping eDNA clones is an important step toward being able to functionally study larger natural product gene clusters from uncultured bacteria. © 2010 Wiley Periodicals, Inc. Biopolymers 93: 833–844, 2010.

## INTRODUCTION

Cultured soil bacteria have been a productive source of both biologically active and structurally diverse natural products.^1^, ^2^ Molecular phylogenetic analysis of soil microbiomes now indicate that a single gram of soil can contain thousands of unique bacterial species, only a small fraction of which is regularly cultured in the laboratory.^3–6^ Uncultured bacteria represent one of the largest pools of genetic diversity that has not been examined for the production of natural products. Culture-independent analysis of microbial communities using DNA extracted directly from environmental samples, which is commonly defined as metagenomics, has the potential to provide access to the biosynthetic capacity of uncultured bacteria.^7^

**FIGURE 1:**
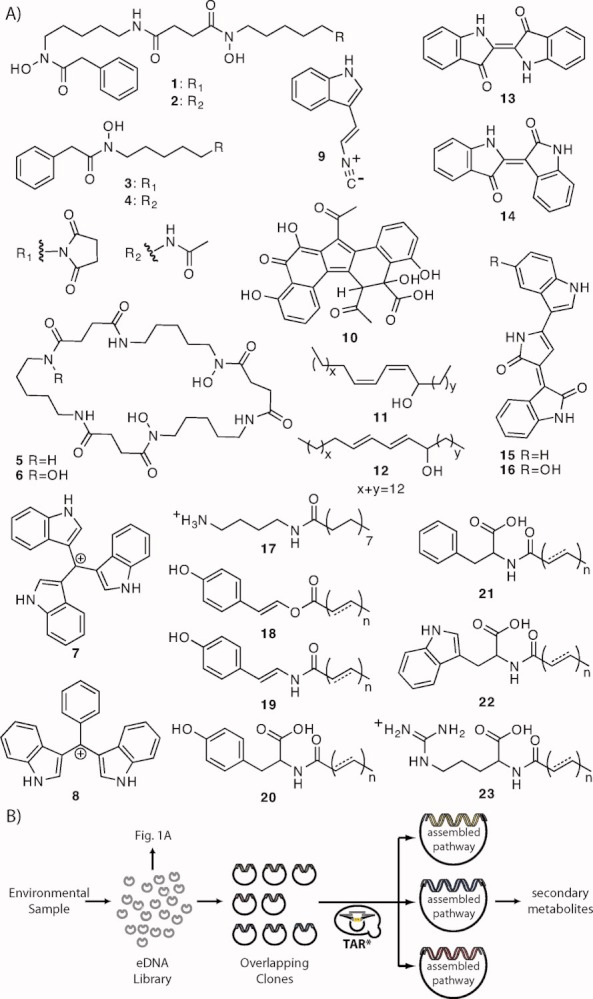
(A) Natural products that have been isolated from individual soil derived eDNA clones are shown. These include terragines A–E (1–5)^8^, norcardamine (6)^8^, turbomycin A (7) and B (8)^9^, a C3-isocyanide functionalized indole derivative (9)^10^, erdacin (10)^11^, aliphatic dienic alcohol isomers (11,12)^12^, indirubin (13)^13^, ^14^, indigo (14)^13^, ^14^, deoxyviolacein (15)^15^, violacein (16)^15^, palmitoylputrescine (17)^16^, long chain enol esters (18)^17^, long chain eneamides (19)^17^, and various long chain *N*-acyl amino acids (20–23).^18–20^ (B) TAR-based gene cluster reassembly strategies can provide access to larger natural product gene clusters captured on overlapping eDNA clones.

All of the genes required for the biosynthesis of a natural product, including genes that encode biosynthetic, regulatory, and self-immunity enzymes, are typically clustered on bacterial chromosomes. Natural product gene clusters can range in size from a few kilobases to over 100 kilobases. The heterologous expression of natural product biosynthetic gene clusters captured on individual eDNA clones has begun to provide access to some of the natural products encoded in the genomes of uncultured bacteria (see Figure 1). However, a major limitation of this strategy has been the inability to routinely construct very large eDNA libraries with inserts big enough to capture large biosynthetic pathways on individual clones. Figure 1 shows a collection of metabolites that have been isolated from the culture broths of soil-derived eDNA clones. In each case, a single cosmid/fosmid eDNA clone confers the production of the metabolites to a heterologous host. Successful functional metagenomic natural product discovery studies carried out on marine samples and other microbiomes have also largely been restricted to single clones.^13^, ^21^

While the construction of 30–40 kb insert cosmid libraries from environmental samples is now routine, the construction of larger insert libraries that can be used to capture large natural product gene clusters has been challenging. Bacterial artificial chromosome (BAC)-derived libraries are capable of capturing larger inserts but generally yield metagenomic libraries that are two to three orders of magnitude smaller than those constructed using cosmid-based cloning strategies.^22^ Theoretically, all gene clusters that are too large to be captured on a single cosmid-sized clone can be reassembled from collections of overlapping eDNA cosmid clones (Figure 1b). Existing gene cluster assembly strategies depend on either unique restriction sites or λ-mediated recombination to reassemble large DNA fragments. Both of these strategies are technically challenging when working with very large DNA fragments or with sequences that span more than two overlapping clones.^11–17^ Transformation-associated recombination (TAR) in *Saccharomyces cerevisiae* relies on homologous recombination to selectively capture a known sequence from a mixture of genomic DNA.^18^, ^19^ In TAR cloning protocols, genomic DNA and a “capture” vector with short homology arms corresponding to sequences flanking the region of interest are cotransformed into *S. cerevisiae*. The capture vector arms and homologous target DNA undergo recombination to yield a stable plasmid containing the targeted genomic region. TAR was originally developed to facilitate cloning large genomic fragments without having to construct and screen genomic DNA libraries. Recent studies extended the scope of this methodology by showing that it could be used to assemble 25 cotransformed overlapping DNA fragments into a complete 592-kb synthetic genome and that multiple PCR products could be assembled into small biochemical pathways.^20–22^ These studies led us to believe that TAR could also be used to assemble large natural product gene clusters from multiple overlapping eDNA clones.

In this report, we show that TAR in *S. cerevisiae* can be used to rapidly reassemble large natural product biosynthetic gene clusters from overlapping eDNA cosmid clones. The rich microbial diversity present in soils makes them attractive, but challenging, starting points for the culture-independent discovery of new natural product biosynthetic gene clusters. Much of the difficulty in working with soil-derived eDNA libraries stems from their inherent complexity, which necessitates the construction of very large clone libraries to ensure that large biosynthetic pathways can be recovered in their entirety. Using two of the largest soil eDNA cosmid libraries reported to date as examples, we have also empirically investigated the minimum size eDNA libraries will likely need to be to recover complete large natural product gene clusters on overlapping cosmid clones. Taken together, these studies provide an experimental framework for gaining access to large, intact natural product biosynthetic gene clusters from soil microbiomes.

## MATERIALS AND METHODS

### Library Construction and Formatting

Top soil collected in Utah and California was used to construct cosmid-based eDNA libraries following methods previously described.^23^ Briefly, the soil was incubated at 70°C in lysis buffer [2% sodium dodecyl sulfate (w/v), 100 m*M* Tris-HCl, 100 m*M* ethylenediaminetetraacetic acid (EDTA), 1.5 *M* NaCl, 1% cetyl trimethylammonium bromide (w/v)] for 2 h. Large particulates were then removed by centrifugation (4,000 × *g*, 30 min). DNA was precipitated from the resulting supernatant with the addition of 0.6 volumes of isopropyl alcohol, pelleted by centrifugation (4000 × *g*, 30 min), washed with 70% ethanol and resuspended in a minimum volume of TE (10 m*M* Tris, 1 m*M* EDTA, pH 8). High molecular weight DNA that was purified from the crude extract by gel electrophoresis (1% agarose, 0.5 × Tris/Borate/EDTA, 16h, 20 V) was blunt-ended (End-It, Epicentre Biotechnologies), ligated into precut pWEB or pWEB-TNC (Epicentre Biotechnologies), packaged into lambda phage, and transduced into *Escherichia coli* (EC100, Epicentre Biotechnologies). Individual library aliquots equivalent to ∼4000–5000 colony forming units were either plated on agar plates or inoculated into 5 ml of liquid LB and then allowed to incubate overnight at 37°C with the appropriate selection. Once colonies formed, the plate-grown aliquots were resuspended in 5 ml of LB. Matching glycerol stocks (15% glycerol) and DNA miniprep pairs were created from each unique library aliquot. The minipreps were arrayed in 8 × 8 grids corresponding to 250,000–320,000 total cosmid clones and DNA from the rows and columns of each grid was pooled. To facilitate library screening, pooled rows and columns were further combined to yield master aliquots, each representing a single 8 × 8 grid of minipreps. Each unique *E. coli* transduction yielded three master aliquots (∼750,000 clones) of the Utah library and one master aliquot (∼320,000 clones) of the California library. In total, the Utah soil library contains ∼10 million unique cosmid clones and the California soil library contains ∼15 million unique cosmid clones.

### Library Size Analysis

DNA from each unique *E. coli* transduction reaction was used as a template in PCR reactions with degenerate primers designed to amplify β-Ketoacyl synthase gene sequences (dp:KS_β_, 5′-TTCGGSGGNTTCCAGWSNGCSATG-3′ and dp:ACP, 5′-TCSAKSAGSGCSANSGASTCGTANCC-3′).^11^, ^38^ Each 25-μl PCR reaction contained 50 ng eDNA template, 2.5 μ*M* of each primer, 2 m*M* dNTPs, 1X ThermoPol Reaction Buffer (New England Biolabs), 0.5 U *Taq* DNA polymerase (New England Biolabs), and 5% dimethyl sulfoxide. Reactions were cycled using the following touchdown protocol: initial denaturation (95°C, 2 min), then eight touchdown cycles [95°C, 45 s; 65°C (dt −1°C/cycle), 1 min; 72°C, 2 min], 35 standard cycles (95°C, 45 s; 58°C, 1 min; 72°C, 2 min) and a final extension step (72°C, 2 min).^11^, ^38^ Amplicons of the correct predicted size (∼1.5 kb) were identified by gel electrophoresis, gel purified, and directly sequenced. In total, DNA from seven unique *E. coli* transductions of the Utah library and 20 unique *E. coli* transductions of the California library was examined.

### Identification of Gene Clusters of Interest

PCR reactions with degenerate primers designed to amplify β-ketoacyl synthase gene sequences were used to detect Type II polyketide synthase (PKS) sequences.^11^, ^38^ Degenerate primers designed to detect flavin-dependent halogenases (TyrohalF3: 5′-CGGCTGGTTCTGGTACATCCC-3′, TyrohalR2: 5′-GAACTCGTAGAASACSCCGTACTC-3′) were used to identify the nonribosomal peptide synthetase (NRPS) gene cluster. The FRI gene cluster was identified using primers that recognize conserved sequences in acyl-CoA ligases found in lipopeptide antibiotic gene clusters (DpFrEFWD1: 5′-TSMTSCAGTACACSTCSGG-3′ and DpFrEREV1: 5′-WDGTCGTASGCGAAGTCSG-3′). Type II PKS sequences were amplified using the same PCR conditions outlined for the library size analysis. Flavin-dependent halogenases were amplified using the following PCR conditions: Each 20-μl reaction contained primer added to a final concentration of 2.5 μ*M*, 0.5 μl of eDNA template (∼100 ng), 1× FailSafe Buffer G (Epicentre Biotechnologies), and 1 U of *Taq* DNA polymerase. Reactions were cycled using the following touchdown protocol: initial denaturation (95°C, 2 min); 9 touchdown cycles [95°C, 30 s; 70°C (dt −1°C/cycle), 30 s; 72°C, 30 s], 30 standard cycles (95°C, 30 s; 60°C, 30 s; 72°C, 30 s), and a final extension step (72°C, 5 min). The acyl-CoA ligase homologues were identified using the following reaction conditions: 25 μl reactions contained primer added to a final concentration of 2.5 μ*M*, 0.5 μl of eDNA template (∼100 ng), 1× ThermoPol Buffer, 2 m*M* dNTPs, and 0.5 U of *Taq* DNA polymerase. Reactions were cycled using the following touchdown protocol: initial denaturation (95°C, 2 min); 6 touchdown cycles [95°C, 30 s; 65°C (dt −1°C/cycle), 30 s; 72°C, 30 s], 30 standard cycles (95°C, 30 s; 58°C, 30 s; 72°C, 30 s), and a final extension step (72°C, 2 min). Amplicons of the correct predicted size were gel purified and directly sequenced.

### General Procedure for Clone Recovery

Individual clones were recovered from a 4000–5000-membered sublibrary by plating a 10^−5^ or 10^−6^ dilution of the corresponding glycerol stock into 96-well microtiter plates and screening the diluted cultures by whole-cell PCR with primers designed to recognize amplicons detected in the initial screen. PCR positive wells were then either subjected to a second round of dilution plating or plated directly on LB agar with ampicillin (50 μg ml^−1^) to yield distinct colonies that were screened by whole-cell PCR to identify individual clones of interest. Each recovered cosmid was end-sequenced using vector-specific (pWEB, pWEB-TNC) universal primers [M13(−40) and the T7 promoter]. All clones were fully sequenced using 454 GLX FLX pyrosequencing, assembled using Newbler (Roche), and annotated using Genemark and BLASTX.^39–41^ Gene cluster images were generated using MacVector. The amino acid substrate specificity for each adenylation domain found in the cryptic NRPS gene cluster was predicted using NRPSpredictor.^42^

### pTARa Vector Construction

The yeast *ARSH4* (autonomous replicating sequence), *CEN6* (plasmid maintenance element), and *URA3* markers were obtained from pLLX13 by digestion with EcoRI and HindIII.^23^ After gel purification, the fragment was ligated into similarly digested pCC1-BAC (Epicentre Biotechnologies). The resulting vector was digested with HpaI and ligated to a DraI fragment from pOJ436 containing an origin of transfer (OriT), integrase and apramycin resistance gene.^43^ Transformation into EPI300 *E. coli* (Epicentre Biotechnologies) and selection on chloramphenicol (12.5 μg ml^−1^) and apramycin (50 μg ml^−1^) yielded the capture vector pTARa (TAR-ready BAC with the *Streptomyces* attP integration system, GenBank accession number: GQ452294).

### TAR Cloning

TAR cloning was initially developed to selectively isolate regions of genomes without the need to construct and screen a genomic library.^23^, ^24^, ^32^, ^33^, ^44^ The procedures outlined below describe our adaptation of these methods for the isolation of sequenced natural product gene clusters and the assembly of large natural product biosynthetic gene clusters captured on multiple overlapping eDNA clones.

### Pathway-Specific Capture Vector Construction

The cycloheximide counter selection cassette (CYH2/*bla*) was PCR amplified using pLLX8 as a template following reported protocols.^23^ The cassette was amplified using primers pLLX8/fw/: 5′-TTTTCTAGAACGCGTTTAATTAAAATCTAAAGTATATATGAGTAAAC-3′ and pLLX8/rv/: 5′-CCCTCTAGAGTTAACGTTTAAACAAAAAACGGTGAAAATGGGTGATAG-3′. Each 50-μl reaction contained 1× FailSafe Buffer B (Epicentre Biotechnologies), 2.5 μ*M* of each primer, 100 ng of pLLX8 template, and 1 U of *Taq* DNA polymerase. Reactions were cycled using the following protocol: initial denaturation (95°C, 2 min), 35 standard cycles (95°C, 30 s; 65°C, 30 s; 72°C, 3 min), and a final extension step (72°C, 7 min). The 2.95-kb PCR product was gel purified prior to capture vector assembly (MinElute™, Qiagen). eDNA clone assembly homology arms were PCR amplified in 25-μl reactions containing 100 ng of template cosmid, 2.5 μ*M* of each primer, 1× FailSafe Buffer D (Epicentre Biotechnologies), and 0.5 U *Taq* DNA polymerase. Reactions were cycled using the following protocol: initial denaturation (95°C, 2 min), 35 standard cycles (95°C, 1 min; 60°C, 1 min; 72°C, 1 min), and a final extension step (72°C, 5min). PCR primers for homology arms were designed to contain 40 bp of homology to the pTARa vector and 40 bp of homology to the counter selection cassette.^23^ These homology regions were incorporated to allow pathway-specific capture vector construction using recombination in *S. cerevisiae*.^23^ Upstream homology arm amplification primers contained a sense primer extension: 5′-ATATTACCCTGTTATCCCTAGCGTAACTATCGATCTCGAG-3′, and an antisense primer extension: 5′-CATATATACTTTAGATTTTAATTAAACGCGTTCTAGAAAA-3′, which add 40 bp of homology to pTARa and the counter selection cassette, respectively. The downstream targeting sequence sense primer extension is: 5′-CATTTTCACCGTTTTTTGTTTAAACGTTAACTCTAGAGGG-3′, which provides homology to the counter selection cassette and the antisense primer extension is: 5′-TAACAGGGTAATATAGAGATCTGGTACCCTGCAGGAGCTC-3′, which provides homology to pTARa. Each primer pair was designed to yield a 600- to 900-bp amplicon that acts as a homology arm in a pathway-specific capture vector used for a TAR reassembly reaction.^23^ Cosmids X16 and V48 were used as templates to generate upstream and downstream homology arms for the PKS gene cluster. Cosmids ZA41 and J2 were used as templates to generate upstream and downstream homology arms for the NRPS gene cluster. Cosmids 1679 and 201 were used as templates to generate upstream and downstream homology arms for the FRI gene cluster. About 300 ng of purified *Citrobacter koseri* genomic DNA (MasterPure™ Complete DNA Purification Kit, Epicentre Biotechnologies) was used as a template to generate upstream and downstream homology arms for the colibactin gene cluster (GenBank accession number: AM229678). Each PCR amplified component was gel purified prior to its use in the assembly of a pathway-specific capture vector.

For the assembly of a pathway-specific capture vector, 200 ng of pTARa was linearized with NheI and added to 200 μg of heat denatured single stranded carrier DNA (heated to 95°C for 10 min then kept on ice), 600 ng of CYH2/*bla* counter selection cassette amplicon^23^ and 200 ng of an upstream and downstream homology arm amplicon pair prepared as described above. All components were added to lithium acetate prepared chemically competent CRY1–2 (uracil deficient, *ura*^−^) yeast, plated on synthetic complete (SC) uracil dropout agar (Invitrogen) and incubated at 30°C.^45^ Colonies typically began to appear within 24–48 h. Assembled capture vectors were isolated in bulk by resuspending yeast colonies from a 100 mm SC dropout agar plate in 5 ml of 1 × phosphate buffered saline. Plasmid DNA was isolated from 1 ml of resuspended cells (ChargeSwitch™ Yeast Plasmid Isolation Kit, Invitrogen). About 100 ng of the purified DNA was transformed into electrocompetent EPI300 *E. coli* and plated onLB agar containing ampicillin (100 μg ml^−1^), chloramphenicol (12.5 μg ml^−1^), and apramycin (50 μg ml^−1^) to yield a pathway-specific capture vector containing a counter-selection cassette.

### TAR Cloning and Pathway Assembly

Direct TAR cloning of the colibactin gene cluster from genomic DNA was carried out using reported protocols.^24^, ^44^ For eDNA pathway assembly, each cosmid to be used in an assembly reaction was initially linearized by digestion with DraI and the capture vector was linearized by digestion with PmeI. About 200 ng of each linearized cosmid and an equimolar amount (∼100 ng) of a linearized pathway-specific capture vector were added to 200 μl of *S. cerevisiae* spheroplasts prepared as previously reported.^44^ The transformed spheroplasts were added to 7 ml of top agar equilibrated to 50°C [1*M* sorbitol, 1.92 g l^−1^ SC uracil dropout supplement (Invitrogen), 6.7 g l^−1^ yeast nitrogen base (Invitrogen), 2% glucose, 2.5% agar]. The top agar containing transformed spheroplasts was overlaid onto SC dropout agar containing 2.5 μg ml^−1^ cycloheximide. The plates were incubated at 30°C and spheroplast growth was typically seen within 72 hours. The resulting recombinants were patched onto SC uracil dropout agar with cycloheximide (2.5 μg ml^−1^) for overnight growth at 30°C.

For initial PCR detection of reassembled pathways, a small portion of each yeast patch was resuspended in 10 μl of 20 m*M* NaOH and heated at 95°C for 10 min. 1.5 μl of the cell lysate was then used as a template in a 50-μl multiplex PCR reaction following the manufacturer's directions (Multiplex PCR Kit, Q solution™, Qiagen). The primer sets used in this analysis were designed to recognize unique regions from each overlapping cosmid clone that was used in an assembly reaction. In the colibactin TAR experiment, PCR primer pairs were designed to detect the previously reported boundaries of the biosynthetic gene cluster.^46^

### Analysis of TAR Recombined Clones

Yeast recombinants that produced PCR amplicons of correct size for all portions of a pathway were grown overnight (30°C, 225 rpm) in 2 ml of SC uracil dropout media (or on SC uracil dropout agar) with 2.5 μg ml^−1^ cycloheximide and TAR assembled pathways were isolated from these cultures (ChargeSwitch™, Invitrogen). Five microliters of ChargeSwitch™ prepared DNA (1/10 elution volume) was transformed into electrocompetent EPI300 *E. coli* which were outgrown at 30°C for 2 h (225 rpm) and then plated on LB agar with 12.5 μg ml^−1^ chloramphenicol. Whole-cell PCR was used to identify *E. coli* colonies containing correctly reassembled gene clusters. DNA was then isolated from 5 ml cultures of PCR positive *E. coli* transformants using alkaline lysis and isopropanol precipitation (CopyControl™ pCC1-BAC Induction Protocol, Epicentre Biotechnologies). *E. coli* transformants containing the colibactin gene cluster were identified using eight sets of previously reported PCR primers designed to detect different ORF's in the pathway (data not shown).^46^ Detailed restriction mapping was carried out on each reassembled pathway using an enzyme (PKS, EcoRI; NRPS, EcoRI; FRI, BglII; Colibactin, HindIII) that was predicted to yield restriction fragments that could be easily resolved using agarose gel electrophoresis (1% agarose, 0.5× Tris/Borate/EDTA, 30 V, overnight). The lambda HindIII and 50-bp molecular weight makers were obtained from New England Biolabs. Full pathway sequencing for each gene cluster was deposited with GenBank under the following accession numbers: NRPS: GQ475282, FRI: GQ475284, and PKS: GQ475283.

### Conjugation and Preparation for Heterologous Expression

Assembled pathways were transformed into S17-1 *E. coli* for conjugation into *Streptomyces* using published protocols.^43^ All three reassembled eDNA gene clusters were successfully conjugated and chromosomally integrated into a number of *Streptomyces* including *Streptomyces lividans, Streptomyces albus*, and *Streptomyces toyocaensis*.^47^

## RESULTS AND DISCUSSION

### Library Size Analysis

The genes responsible for the biosynthesis of a natural product are typically clustered on a bacterial chromosome, and therefore theoretically can be cloned on a single continuous fragment of eDNA. While the heterologous expression of biosynthetic gene clusters captured on eDNA-derived clones has begun to yield novel natural products, many natural product gene clusters are too large to be routinely captured on individual eDNA cosmid clones (see Figure 1). With metagenomic libraries of sufficient size and sequence coverage, large gene clusters that cannot be captured on a single cosmid clone could be accessed by recovering collections of overlapping eDNA clones. Soil microbiomes are among the most genetically diverse environments characterized to date and are therefore attractive starting points for the discovery of natural products using a metagenomic approach.^3^ However, because of this complexity, it is difficult to predict the size a soil-based eDNA library must be to permit the recovery of overlapping clones from a diverse collection of large natural product gene clusters. We set out to empirically investigate this problem using eDNA libraries constructed from two different soil samples. For this study, DNA isolated from soil collected in Utah was used to construct a series of independent 750,000-membered eDNA cosmid libraries (∼10,000,000 clones in total) and DNA isolated from a soil sample collected in California was used to construct a series of independent 320,000-membered eDNA cosmid libraries (∼15,000,000 clones in total).

**FIGURE 2:**
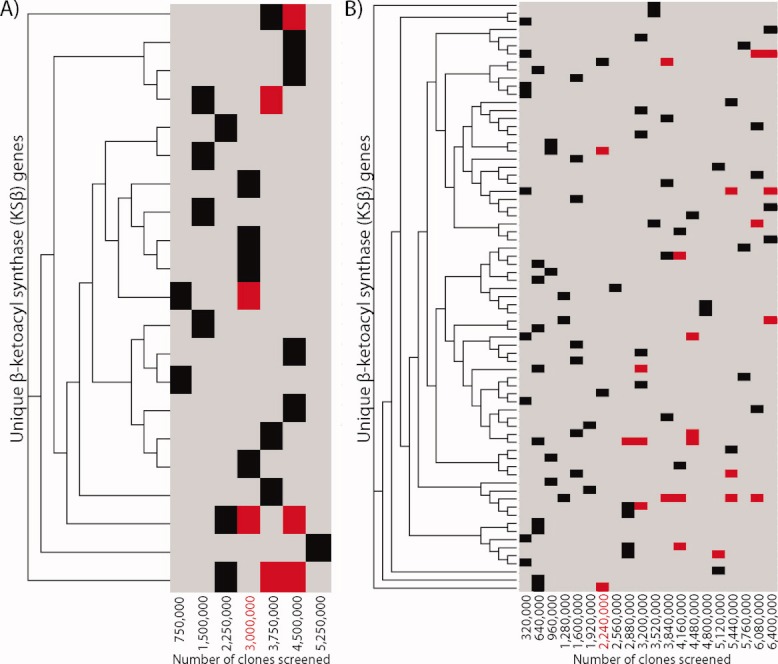
Degenerate primers targeting minimal Type II PKS genes were used to identify KS_β_ sequences present in unique eDNA library aliquots constructed from soil samples collected in Utah (A) and California (B). ClustalW^48^ derived phylogenetic trees of the KS_β_ sequences identified in these screens are shown. The aliquots from which sequences were amplified and the point at which they began to reappear in the library (red) are shown as a heatmap.

The reassembly of large natural product gene clusters from multiple overlapping eDNA fragments begins with the detection of specific sequence(s) of interest located on two or more unique library clones. We therefore wanted to determine the point at which redundant sequences of interest began to regularly appear in unique eDNA library aliquots constructed from the same soil sample. Culture-based studies suggest that Type II (aromatic, iterative) PKS biosynthetic systems are common in bacteria and the PKS genes found in these systems are highly conserved. We therefore chose Type II PKS pathways as a model system for studying large (>30 kb) gene clusters present in soil-derived eDNA libraries. Both the California and Utah libraries were screened for the presence of β-ketoacyl synthase (KS_β_) gene sequences using degenerate PCR primers designed to recognize Type II PKS systems.^11^, ^38^ In total, 19 distinct KS_β_ gene sequences were amplified from the Utah library and 73 distinct KS_β_ gene sequences were amplified from the California library (see Figure 2). In the Utah library, redundant KS_β_ sequences began to regularly appear once ∼3 × 10^6^ clones had been examined, while in the California library redundant KS_β_ sequences began to regularly appear once ∼2.25 × 10^6^ clones had been examined. Additional screens using primers designed to recognize other conserved natural product biosynthetic gene sequences have shown similar results. In these studies, redundant sequences begin to regularly appear once libraries exceed 1–3 × 10^6^ clones in size (data not shown).^49^ The libraries used in our efforts to recover natural product gene clusters to be used in TAR assembly experiments were therefore expanded until they contained at least 1–1.5 × 10^7^ unique clones, which corresponds to 5–10 times the number of clones needed to identify the first redundant Type II PKS sequences. While even an eDNA library of 1–1.5 × 10^7^ clones is unlikely to permit the recovery of rare gene clusters, our analysis suggests that it will likely contain collections of clones encompassing complete PKS gene clusters and, by extension, overlapping clones from many other types of biosynthetic gene clusters found in the genomes of uncultured bacteria.

### Natural Product Gene Cluster Identification and Recovery

In excess of 35,000 unique microbial natural products have been characterized using culture-based methods.^50^, ^51^ This amazing assortment of natural products is biosynthesized using a much smaller number of conserved enzyme families. The structural diversity seen in natural products appears to arise in large part from the natural combinatorial shuffling of these conserved biosynthetic enzyme families.^52^ Degenerate primers designed to recognize conserved natural product biosynthetic gene sequences should therefore be useful for identifying eDNA derived gene clusters that encode the biosynthesis of a diverse collection of small molecules. In this study, three different sets of degenerate primers were used to recover three large natural product biosynthetic gene clusters from the Utah and California soil eDNA libraries. A cryptic Type II PKS gene cluster was identified using the Type II PKS-specific degenerate primers we used in our initial library size analysis.^11^, ^38^ A cryptic NRPS gene cluster was identified using degenerate primers designed to amplify flavin-dependent halogenases known to tailor aromatic amino acids found in halogenated nonribosomal peptides. Degenerate primers designed to recognize acyl-CoA ligases found in lipopeptide antibiotic gene clusters were used to identify a gene cluster that is predicted to encode the known metabolite friulimicin. These three eDNA-derived gene clusters are referred to as the PKS, NRPS and FRI gene clusters, respectively. The PKS and NRPS gene clusters were found in an eDNA library derived from topsoil collected in Utah while the FRI gene cluster was found in an eDNA library derived from desert soil collected in California.

**FIGURE 3:**
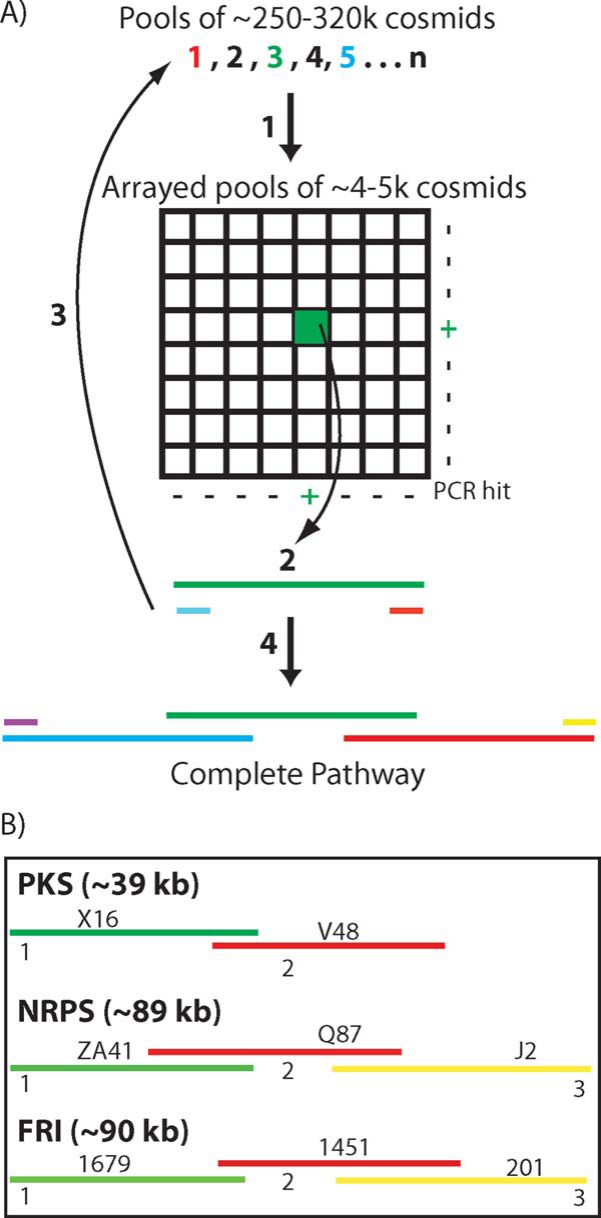
(A) PCR with degenerate primers was used to identify biosynthetic genes of interest in large library pools (1) and then to subsequently locate these same sequences in arrays of smaller library aliquots (+). Whole cell PCR of serially diluted smaller library aliquots was used to recover individual cosmids of interest (2). Overlapping clones were iteratively recovered (3) until complete biosynthetic pathways were identified (4). (B) The topology of the overlapping clones that are predicted to comprise the eDNA derived PKS, NRPS, and FRI gene clusters is shown.

Individual cosmid clones containing genes recognized by the degenerate primers used in initial library screens were recovered from the appropriate library and then end sequenced (see Figure 3). PCR primers designed against the end sequences were subsequently used to identify and recover overlapping clones from the same library. The process of clone recovery and end sequencing was iteratively repeated until genes predicted to be involved in primary metabolism were found on the distal ends of a recovered cosmid (see Figure 3). This initial end-sequencing analysis suggested that the NRPS and FRI gene clusters were recovered on three cosmids each (NRPS: clones ZA41, Q87, J2; FRI: clones 1697, 1451, 201). The PKS gene cluster appeared to be present on two overlapping cosmids (PKS: clones X16, V48).

Each clone that was predicted to be part of a gene cluster was fully sequenced and annotated. The eDNA-derived FRI gene cluster and the friulimicin gene cluster from *A. friuliensis* have the same gene organization and are 89% identical over the 68-kb region that is predicted to comprise the functional biosynthetic pathway.^53^ A comparison of these two gene clusters suggests that the entire FRI gene cluster was likely captured on the three overlapping eDNA cosmids that were recovered. While the eDNA-derived PKS and NRPS gene clusters do not closely resemble any known gene clusters, the appearance of primary metabolic enzymes in the sequence surrounding the conserved natural product biosynthetic genes found on these clones suggests they were also likely recovered in their entirety. Sequencing of a fourth overlapping clone that extends 20 kb beyond the NRPS gene cluster found no enzymes associated with secondary metabolism. As suggested by our initial eDNA library size analysis, cosmid libraries containing in excess of 10 million clones appear to provide sufficient coverage of soil metagenomes to allow access to a diverse range of complete natural product biosynthetic gene clusters.

### TAR Vector Design and Construction

**FIGURE 4:**
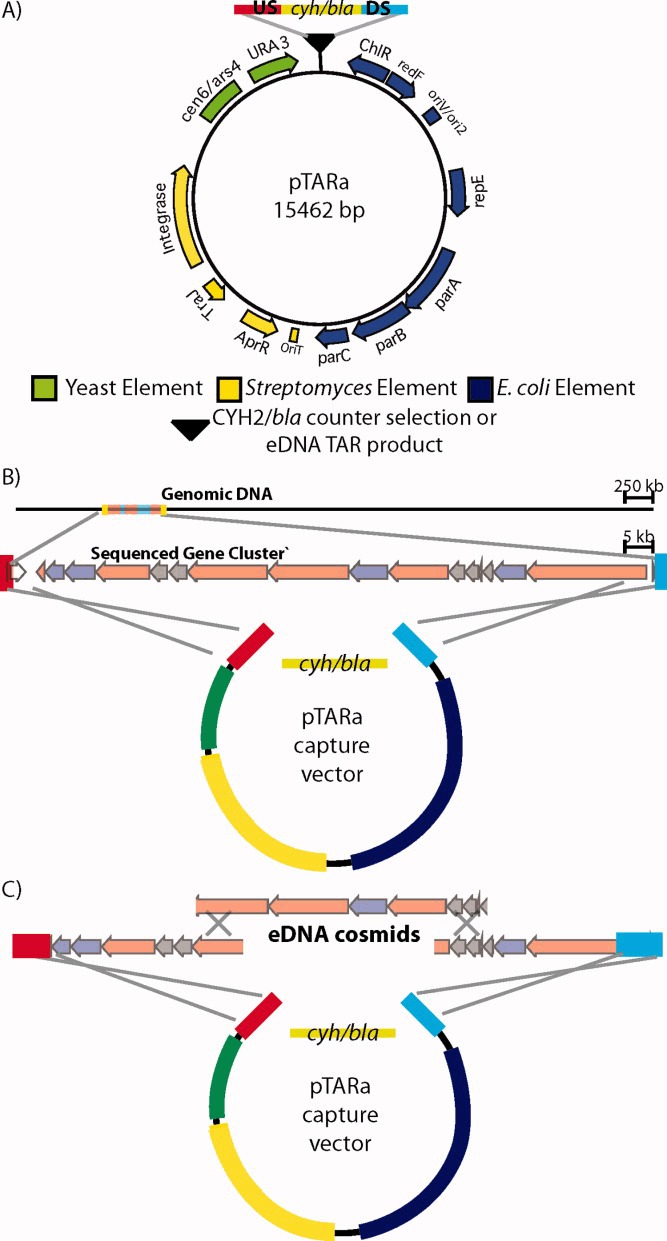
(A) pTARa contains elements that allow for the rapid assembly and propagation of pathways in *S. cerevisiae* (green), the transformation and analysis of these pathways in *E. coli* (blue) and the integrative conjugation of assembled pathways into *Streptomyces* (yellow). For capture vector construction, pathway-specific upstream (US-blue), and downstream (DS-red) homology arms, as well as a counter selection cassette (*cyh/bla*) are incorporated into the capture vector.^23^, ^24^ During recombination, the counter selection cassette is exchanged for a TAR cloned gene cluster (B) or TAR reassembled eDNA pathway (C).

To facilitate TAR reassembly of large natural product gene clusters as well as subsequent heterologous expression studies with reassembled pathways, we created pTARa, a BAC-based *S. cerevisiae/E. coli/Streptomyces* shuttle capture vector (Figure 4a). This vector contains elements that allow pathways to be assembled in *S. cerevisiae*, characterized and maintained in *E. coli*, and conjugatively transferred into a wide range of *Streptomycetes* for heterologous expression studies.^43^ We included these elements to facilitate *Streptomyces*-based heterologous expression studies, but any number of species-specific genetic elements can be incorporated into pTARa to allow the transfer of pathways into a wide variety of bacterial hosts.^23^ As a demonstration of the utility of pTARa as a shuttle vector, we propagated the vector in *S. cerevisiae* (CRY1–2), transformed and isolated the vector from *E. coli* and successfully conjugated into a number of different *Streptomycetes* including *S. toyocaensis, S. lividans*, and *S. albus*.

### Capturing Natural Product Gene Clusters from Sequenced Genomes Using pTARa

**FIGURE 5:**
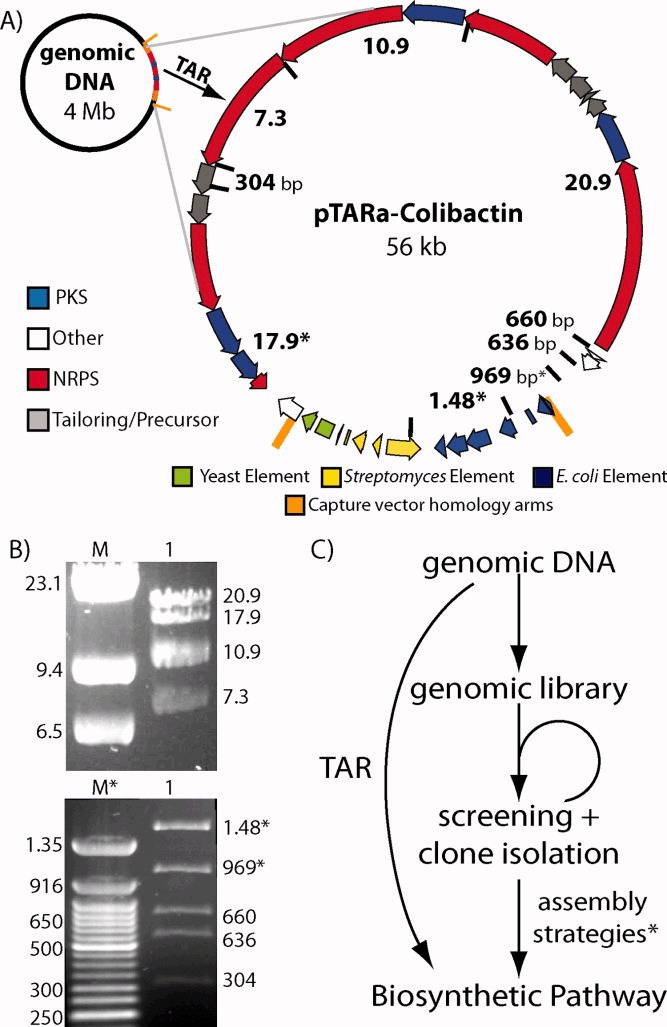
(A) We used pTARa to directly and specifically clone the colibactin gene cluster from *C. koseri* genomic DNA. Predicted HindIII cut sites and restriction fragment sizes are marked on the map of the pTARa-Colibactin construct. The size of the gene cluster is listed. (B) The experimentally determined HindIII restriction map of pTARa-Colibactin is shown. Two images of the same digest were taken at different points during electrophoresis to highlight fragment sizes more clearly (M = Lambda HindIII digest, M* = 50 bp ladder). (C) TAR cloning of gene clusters circumvents the need to construct and screen a genomic library.

The cloning of natural product gene clusters from cultured organisms traditionally requires the construction and screening of a genomic DNA library.^54^, ^55^ Using TAR cloning, a sequenced biosynthetic gene cluster of any size can be directly cloned without the need to construct or screen a genomic library (Figure 5c).^23^ To demonstrate the utility of pTARa for culture-based natural products research, we directly cloned the 56-kb colibactin gene cluster directly from genomic DNA isolated from the cultured bacterium, *C. koseri*.^24^, ^44^ Previous studies determined the functional boundaries of the colibactin gene cluster via transposon mutagenesis.^46^ In order to TAR clone this gene cluster, we simply designed a pathway-specific capture vector using this information (Figures 4b and 5), and co-transformed the capture vector and *C. koseri* genomic DNA into *S. cerevisiae* spheroplasts.^24^, ^44^ We screened yeast spheroplasts using colibactin gene cluster specific PCR primers and were able to quickly identify clones containing intact colibactin gene clusters. Detailed restriction mapping of the TAR cloned pathway confirmed that we had specifically cloned the colibacin gene cluster (pTARa-Colibactin) directly from *C.koseri* genomic DNA (Figure 5b).^46^ As demonstrated by this experiment, TAR cloning should provide a general and rapid means to access intact natural product biosynthetic gene clusters from sequenced microorganisms without the need to construct or screen a genomic library (Figure 5c).

### TAR Assembly of Multiclone Gene Clusters

**FIGURE 6:**
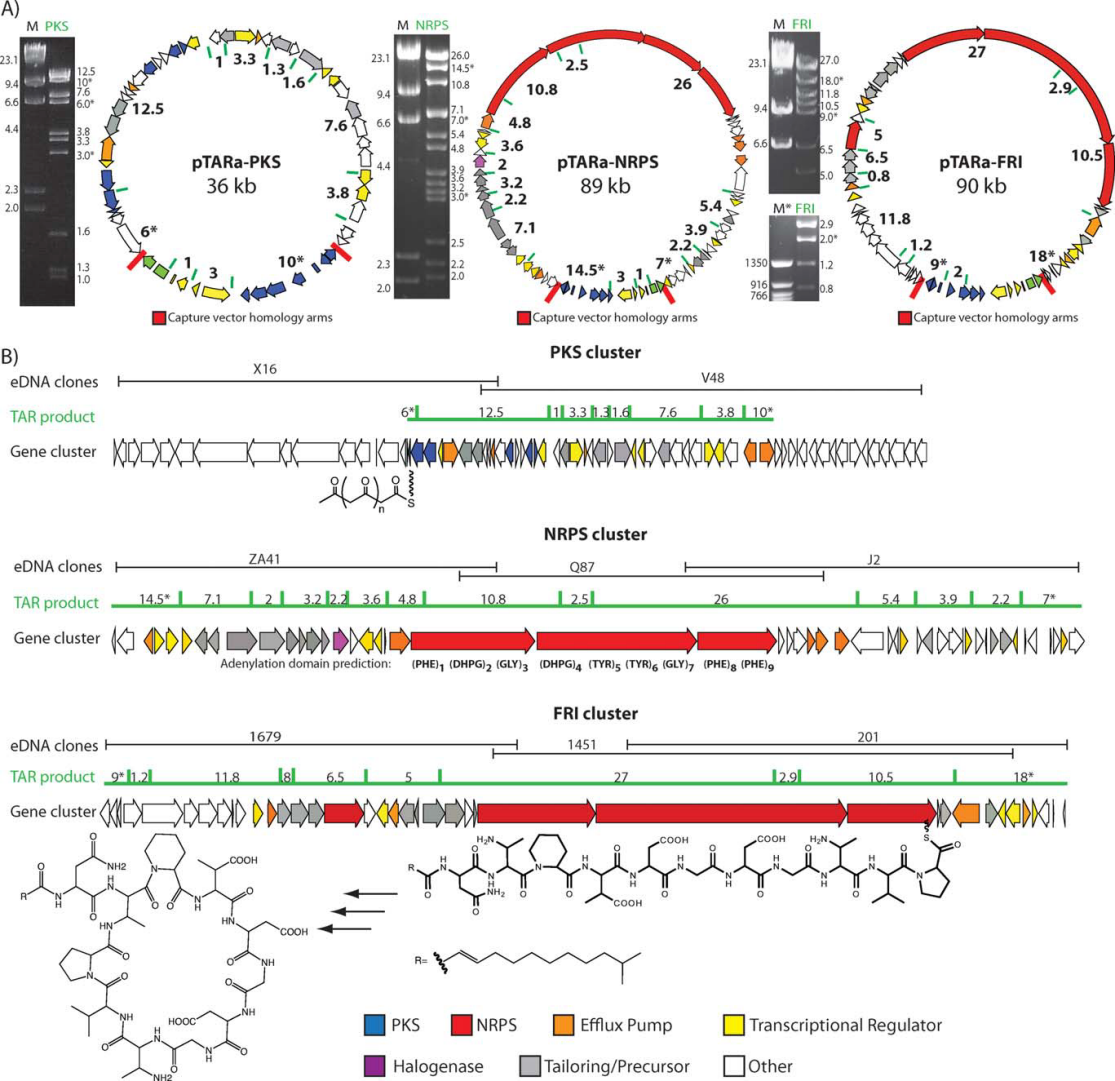
A) Experimentally determined restriction maps and predicted restriction enzyme cut sites for each reconstructed gene cluster are shown. The size of each gene cluster is listed for clarity. (M = Lambda HindIII digest, M* = 50 bp ladder). B) The overlapping cosmids (black) comprising a complete biosynthetic pathway is shown above the region targeted for TAR assembly (green line). The individual building blocks that are predicted to be used by the conserved modules (PKS and NRPS) found in these biosynthetic pathways appear below each gene cluster (DHPG = dihydroxyphenylglycine).^42^

For each reassembly experiment, we constructed a unique pathway-specific capture vector with homology arms corresponding to sequences at the proximal and distal ends of the gene cluster to be reassembled (Figures 4c and 6). Homologous recombination in *S. cerevisiae* is stimulated by the presence of double stranded breaks adjacent to recombination sites.^32^ The individual cosmids to be used in the reassembly of a gene cluster were therefore linearized by restriction digestion with DraI and then cotransformed with a linearized pathway-specific capture vector into competent CRY1–2 *S. cerevisiae*. DraI, which recognizes the AT rich hexamer, TTTAAA, digests the cosmid backbone, yet rarely cuts in GC rich sequences found in biosynthetic gene clusters thus providing a means to generate linear DNA fragments for TAR reassembly reactions. The concentration of the components used in the cotransformation step was empirically determined and selected to yield, on average, one assembled construct per spheroplast. After 3–5 days of recovery on SC uracil dropout agar, recovered spheroplasts were restruck on new SC uracil dropout agar plates. This step is necessary to reduce the chance of cross contamination caused by DNA from the TAR reaction during the PCR analysis that is used to identify yeast colonies with assembled gene clusters. Yeast colonies were then screened using multiplex PCR with primers specific to each unique cosmid fragment predicted to be present in a reassembled gene cluster construct. Between 30 and 70% of the yeast colonies were found to be PCR positive for all fragments predicted to be present in a pathway. Using this approach we were able to rapidly identify yeast colonies that contained intact biosynthetic gene clusters.

Large constructs isolated from PCR positive yeast clones were electroporated into *E. coli* and analyzed by detailed restriction analysis (see Figure 6). In each case, the large construct obtained from a TAR reassembly reaction produced a restriction map that was identical to the map predicted to arise from assembling the individual overlapping clones used in the reaction (see Figure 6). The 39 kb PKS gene cluster was successfully subcloned from the central region of cosmids X16 and V48, two cosmids that contain 2.1 kb of overlap. The entire 89-kb cryptic NRPS gene cluster was successfully reconstructed in a single *S. cerevisiae* spheroplast transformation reaction from three overlapping eDNA cosmid clones. In a similar fashion, we reassembled the 90-kb eDNA-derived FRI gene cluster using a single *S. cerevisiae* spheroplast transformation reaction and three overlapping eDNA-derived cosmid clones.

While the PKS and NRPS gene clusters were initially assembled from fully sequenced sets of cosmids, reassembly experiments can also be performed in the absence of comprehensive sequencing. The FRI gene cluster was originally reassembled with only end-sequencing data for each cosmid clone predicted to comprise the complete gene cluster (Figures 4c and 6). A capture vector based on the end-sequencing data from the distal ends of the two outermost clones, cosmids 1679 and 201, was used to reassemble the gene cluster (see Figure 3). We confirmed the successful reassembly of the fragments using PCR and by comparing restriction maps of the reassembled construct with those produced by the cosmids used in the reassembly experiment (data not shown). Subsequent full sequencing of the clones comprising the FRI gene cluster confirmed the restriction mapping and successful sequencing-independent TAR assembly experiment (see Figure 6).

Traditional gene cluster assembly strategies can become technically impractical when working with large naturally derived DNA sequences. Unique and conveniently located restriction sites needed for traditional “cut and paste” strategies are often not available when working with long natural DNA sequences. Recently, λ-based recombination has been used to reconstruct functional gene clusters, circumventing many of the problems associated with traditional strategies.^26^ Lambda-based recombination becomes difficult, however, for large gene clusters captured on multiple overlapping clones because it requires the step-wise recombination of two clones at a time. This step-wise recombination process requires the introduction of a unique selectable marker into each fragment used in an assembly experiment. As demonstrated here, TAR-dependent assembly of multiclone natural product gene clusters can be performed in a single reaction without any of these barriers. The maximum number of DNA fragments that can be simultaneously assembled in TAR experiments has yet to be determined, but even the largest gene clusters are unlikely to require more than three or four overlapping cosmids which is well within the established limits of TAR.^34^, ^35^, ^56^

## CONCLUSIONS

Previous studies have demonstrated that metagenomic strategies can be used to uncover metabolites encoded by gene clusters captured on individual soil-derived eDNA clones (see Figure 1). Cloning large natural product gene clusters presents a challenge for both culture dependent and culture independent studies. We have shown that TAR can be used to rapidly reassemble overlapping eDNA-derived clones into a single construct containing large eDNA derived natural product gene clusters. We have also shown that TAR can be used to directly and specifically clone natural product gene clusters from sequenced organisms without constructing and screening a genomic library. TAR-dependent assembly of natural product gene clusters from overlapping clones found in eDNA soil-libraries provides an experimental framework for rapidly accessing intact natural product gene clusters that exceed conventional eDNA cloning limits (Figure 1b). In doing so, it eliminates one of the major roadblocks associated with current metagenomic natural product discovery efforts. In this study, this experimental approach provided access to both a new example of what was thought to be a rare gene cluster (FRI) as well as what appear to be new gene clusters (PKS, NPRS). The heterologous expression of large TAR-assembled gene clusters should form a basis for the identification of new natural products from eDNA. The major remaining challenge to the discovery of new natural products from uncultured bacteria, that of heterologous expression, is not unique to culture-independent studies and will likely need to be addressed using many different gene cluster specific strategies.

## References

[1] Newman DJ, Cragg GM (2004). J Nat Prod.

[2] Newman DJ, Cragg GM (2007). J Nat Prod.

[3] Gans J, Wolinsky M, Dunbar J (2005). Science.

[4] Rappe MS, Giovannoni SJ (2003). Annu Rev Microbiol.

[5] Torsvik V, Goksoyr J, Daae FL (1990). Appl Environ Microbiol.

[6] Torsvik V, Ovreas L, Thingstad TF (2002). Science.

[7] Handelsman J, Rondon MR, Brady SF, Clardy J, Goodman RM (1998). Chem Biol.

[8] Wang GY, Graziani E, Waters B, Pan W, Li X, McDermott J, Meurer G, Saxena G, Andersen RJ, Davies J (2000). Org Lett.

[9] Gillespie DE, Brady SF, Bettermann AD, Cianciotto NP, Liles MR, Rondon MR, Clardy J, Goodman RM, Handelsman J (2002). Appl Environ Microbiol.

[10] Brady SF, Clardy J (2005). Angew Chem Int Ed Engl.

[11] King RW, Bauer JD, Brady SF (2009). Angew Chem Int Ed Engl.

[12] Courtois S, Cappellano CM, Ball M, Francou FX, Normand P, Helynck G, Martinez A, Kolvek SJ, Hopke J, Osburne MS, August PR, Nalin R, Guerineau M, Jeannin P, Simonet P, Pernodet JL (2003). Appl Environ Microbiol.

[13] Guan C, Ju J, Borlee BR, Williamson LL, Shen B, Raffa KF, Handelsman J (2007). Appl Environ Microbiol.

[14] Lim HK, Chung EJ, Kim JC, Choi GJ, Jang KS, Chung YR, Cho KY, Lee SW (2005). Appl Environ Microbiol.

[15] Brady SF, Chao CJ, Handelsman J, Clardy J (2001). Org Lett.

[16] Brady SF, Clardy J (2004). J Nat Prod.

[17] Brady SF, Chao CJ, Clardy J (2002). J Am Chem Soc.

[18] Brady SF, Clardy J (2005). Org Lett.

[19] Brady SF, Chao CJ, Clardy J (2004). Appl Environ Microbiol.

[20] Clardy J, Brady SF (2007). J Bacteriol.

[21] Schmidt EW, Nelson JT, Rasko DA, Sudek S, Eisen JA, Haygood MG, Ravel J (2005). Proc Natl Acad Sci USA.

[22] Liles MR, Williamson LL, Rodbumrer J, Torsvik V, Goodman RM, Handelsman J (2008). Appl Environ Microbiol.

[23] Mathee K, Narasimhan G, Valdes C, Qiu X, Matewish JM, Koehrsen M, Rokas A, Yandava CN, Engels R, Zeng E, Olavarietta R, Doud M, Smith RS, Montgomery P, White JR, Godfrey PA, Kodira C, Birren B, Galagan JE, Lory S (2008). Proc Natl Acad Sci USA.

[24] Kouprina N, Larionov V (2008). Nat Protoc.

[25] Zhang Y, Buchholz F, Muyrers JP, Stewart AF (1998). Nat Genet.

[26] Wenzel SC, Gross F, Zhang Y, Fu J, Stewart AF, Muller R (2005). Chem Biol.

[27] Holt RA, Warren R, Flibotte S, Missirlis PI, Smailus DE (2007). Bioessays.

[28] Sharan SK, Thomason LC, Kuznetsov SG, Court DL (2009). Nat Protoc.

[29] Thomason L, Court DL, Bubunenko M, Costantino N, Wilson H, Datta S, Oppenheim A (2007). Curr Protoc Mol Biol.

[30] Sawitzke JA, Thomason LC, Costantino N, Bubunenko M, Datta S, Court DL (2007). Methods Enzymol.

[31] Court DL, Sawitzke JA, Thomason LC (2002). Annu Rev Genet.

[32] Larionov V, Kouprina N, Eldarov M, Perkins E, Porter G, Resnick MA (1994). Yeast.

[33] Larionov V, Kouprina N, Graves J, Resnick MA (1996). Proc Natl Acad Sci USA.

[34] Gibson DG, Benders GA, Andrews-Pfannkoch C, Denisova EA, Baden-Tillson H, Zaveri J, Stockwell TB, Brownley A, Thomas DW, Algire MA, Merryman C, Young L, Noskov VN, Glass JI, Venter JC, Hutchison CA, Smith HO (2008). Science.

[35] Gibson DG, Benders GA, Axelrod KC, Zaveri J, Algire MA, Moodie M, Montague MG, Venter JC, Smith HO, Hutchison CA (2008). Proc Natl Acad Sci USA.

[36] Shao Z, Zhao H (2009). Nucleic Acids Res.

[37] Brady SF (2007). Nat Protoc.

[38] Seow KT, Meurer G, Gerlitz M, Wendt-Pienkowski E, Hutchinson CR, Davies J (1997). J Bacteriol.

[39] Lukashin AV, Borodovsky M (1998). Nucleic Acids Res.

[40] Margulies M, Egholm M, Altman WE, Attiya S, Bader JS, Bemben LA, Berka J, Braverman MS, Chen YJ, Chen Z, Dewell SB, Du L, Fierro JM, Gomes XV, Godwin BC, He W, Helgesen S, Ho CH, Irzyk GP, Jando SC, Alenquer ML, Jarvie TP, Jirage KB, Kim JB, Knight JR, Lanza JR, Leamon JH, Lefkowitz SM, Lei M, Li J, Lohman KL, Lu H, Makhijani VB, McDade KE, McKenna MP, Myers EW, Nickerson E, Nobile JR, Plant R, Puc BP, Ronan MT, Roth GT, Sarkis GJ, Simons JF, Simpson JW, Srinivasan M, Tartaro KR, Tomasz A, Vogt KA, Volkmer GA, Wang SH, Wang Y, Weiner MP, Yu P, Begley RF, Rothberg JM (2005). Nature.

[41] Tatusov RL, Altschul SF, Koonin EV (1994). Proc Natl Acad Sci USA.

[42] Rausch C, Weber T, Kohlbacher O, Wohlleben W, Huson DH (2005). Nucleic Acids Res.

[43] Bierman M, Logan R, O'Brien K, Seno ET, Rao RN, Schoner BE (1992). Gene.

[44] Kouprina N, Noskov VN, Larionov V (2006). Methods Mol Biol.

[45] Gietz RD, Schiestl RH (2007). Nat Protoc.

[46] Nougayrede JP, Homburg S, Taieb F, Boury M, Brzuszkiewicz E, Gottschalk G, Buchrieser C, Hacker J, Dobrindt U, Oswald E (2006). Science.

[47] Tobias Kieser MJB, Mark JB, Keith FC, David AH (2000). Practical Streptomyces Genetics.

[48] Thompson JD, Gibson TJ, Higgins DG (2002). Curr Protoc Bioinformatics.

[49] Banik JJ, Brady SF (2008). Proc Natl Acad Sci USA.

[50] Buckingham J (2007). Dictionary of Natural Products.

[51] Laatsch H, Laatsch H (2009). AntiBase 2009: The Natural Compound Identifier.

[52] Dewick PM (2002). Medicinal Natural Products: A Biosynthetic Approach.

[53] Muller C, Nolden S, Gebhardt P, Heinzelmann E, Lange C, Puk O, Welzel K, Wohlleben W, Schwartz D (2007). Antimicrob Agents Chemother.

[54] Miao V, Coeffet-Legal MF, Brian P, Brost R, Penn J, Whiting A, Martin S, Ford R, Parr I, Bouchard M, Silva CJ, Wrigley SK, Baltz RH (2005). Microbiology.

[55] McHenney MA, Hosted TJ, Dehoff BS, Rosteck PR, Baltz RH (1998). J Bacteriol.

[56] Gibson DG (2009). Nucleic Acids Res.

